# A Beneficiary Role for Neuraminidase in Influenza Virus Penetration through the Respiratory Mucus

**DOI:** 10.1371/journal.pone.0110026

**Published:** 2014-10-15

**Authors:** Xiaoyun Yang, Lennert Steukers, Katrien Forier, Ranhua Xiong, Kevin Braeckmans, Kristien Van Reeth, Hans Nauwynck

**Affiliations:** 1 Laboratory of Virology, Department of Virology, Parasitology and Immunology, Faculty of Veterinary Medicine, Ghent University, Merelbeke, Belgium; 2 Laboratory of General Biochemistry and Physical Pharmacy, Ghent University, Ghent, Belgium; 3 Center for Nano- and Biophotonics, Ghent University, Ghent, Belgium; Mount Sinai School of Medicine, United States of America

## Abstract

Swine influenza virus (SIV) has a strong tropism for pig respiratory mucosa, which consists of a mucus layer, epithelium, basement membrane and lamina propria. Sialic acids present on the epithelial surface have long been considered to be determinants of influenza virus tropism. However, mucus which is also rich in sialic acids may serve as the first barrier of selection. It was investigated how influenza virus interacts with the mucus to infect epithelial cells. Two techniques were applied to track SIV H1N1 in porcine mucus. The microscopic diffusion of SIV particles in the mucus was analyzed by single particle tracking (SPT), and the macroscopic penetration of SIV through mucus was studied by a virus in-capsule-mucus penetration system, followed by visualizing the translocation of the virions with time by immunofluorescence staining. Furthermore, the effects of neuraminidase on SIV getting through or binding to the mucus were studied by using zanamivir, a neuraminidase inhibitor (NAI), and *Arthrobacter ureafaciens* neuraminidase. The distribution of the diffusion coefficient shows that 70% of SIV particles were entrapped, while the rest diffused freely in the mucus. Additionally, SIV penetrated the porcine mucus with time, reaching a depth of 65 µm at 30 min post virus addition, 2 fold of that at 2 min. Both the microscopic diffusion and macroscopic penetration were largely diminished by NAI, while were clearly increased by the effect of exogenous neuraminidase. Moreover, the exogenous neuraminidase sufficiently prevented the binding of SIV to mucus which was reversely enhanced by effect of NAI. These findings clearly show that the neuraminidase helps SIV move through the mucus, which is important for the virus to reach and infect epithelial cells and eventually become shed into the lumen of the respiratory tract.

## Introduction

Pigs are naturally susceptible to three subtypes of influenza A viruses: H1N1, H3N2 and H1N2, all of which have a strong tropism for the pig respiratory tract mucosa [1]–[3]. Swine influenza virus particles are transmitted by direct contact and through the air in large droplets or as aerosols [1], [4], [5]. During the transmission from pig to pig, the virus first encounters mucus, the first barrier of the respiratory tract. After overcoming this barrier, the virus reaches the target cells in the mucosal epithelium. Influenza virus infects host cells by binding to cellular receptors via one of the major viral glycoproteins, hemagglutinin (HA). HA binds to sialic acids (SA) on the cell surface and mediates the subsequent membrane fusion leading to virus entry [6]. Neuraminidase (NA) catalyzes the removal of terminal sialic acids on the cellular surface to release the progeny virus [7]. It is well documented that the NA functions at the releasing stage of the virus replication [8]–[10], while little is known if NA plays a role during the virus entry into host cells and even less on if it helps the virus overcome the mucus layer.

Mucus is a complex mixture of mucous glycoproteins (mucins), proteins, proteases and protease inhibitors, lipids and water [11], [12]. Mucins, the major component of mucus, are highly o-glycosylated with glycans covalently linked via N-acetylgalactosamine (GaNAc) to the hydroxyl groups of serine and threonine residues of the mucin backbone [12], [13]. Most of the sugar chains of mucin monomers are terminated with sialic acid, which is also known to be the cellular receptor of influenza viruses. It is hypothesized that influenza viruses bind to these extracellular receptors, get entrapped in the mucus and then are removed by ciliary clearance [14]–[16]. Several studies have shown that interaction of influenza virus with mucus results in competitive inhibition of the virus. Roberts *et al.* [17] showed that pre-incubation of human H3N2 virus strain A/Victoria/3/75 with ferret nasal washes containing mucus clearly reduced the virus infectivity, and this inhibition was correlated to competitive binding of the virus with alpha 2,3 and 2,6 linked sialic acids (α2,3- and α2,6-SA) present in the mucus secretions. The protective effect of the mucus barrier was confirmed by a recent study using a transgenic mouse model that overexpressed SA α2-3 Gal rich Muc5AC. Transgenic mice challenged with A/PR8/34 H1N1, which preferentially binds α2,3-SA showed significant less infection than the normal mice [18]. These studies suggest that mucus or mucins block the influenza virus infection by competitively inhibiting HA-mediated cell adsorption.

Despite this inhibitory function of the mucus, the virus is ultimately able to reach the susceptible epithelial cells. It has long been assumed that NA promotes virus access to target cells in the airway by mucus degradation. However, this concept is scarcely supported by experimental data. Cohen *et al.* [19] incubated A/PR/8/34 H1N1 and A/Aichi/2/68 H3N2 virus with human salivary mucins which were previously coated on magnetic beads, and after extensive washings, detected the remained Neu5AC on the mucins. They showed that these human influenza viruses had cleaved away 40–60% of Neu5AC content of the mucins by their viral neuraminidase. The effective cleavage may allow the efficient release of virus from the mucus. This contrasts with the findings of Ehre *et al.* [18] who demonstrated a strong protection of Muc5AC up-regulated mice against A/PR/8/34 H1N1 virus infection. Hence, the purified human salivary mucins may not fully reflect the natural mucus as these mucins had been highly modified after attaching to magnetic beads. Unraveling the mechanism behind the penetration of viruses across the mucosal barriers has potentially significant implications for the development of novel antiviral strategies. Therefore, an *in vitro* model resembling the *in vivo* situation is needed and the interactions of influenza virus with natural mucus should be studied in depth. In the present study, we aimed to address the following questions:

1. Is the virus entrapped or able to penetrate through the native respiratory mucus?
2. Can viral neuraminidase use mucin sialic acids as substrates and catalyze the cleavage of them away?
3. Is this cleavage sufficient to liberate the virions and allow them to penetrate through the mucus layer?

To this purpose, we applied swine influenza virus to a model we previously set up using porcine respiratory mucus, pseudorabies virus (PRV) and single particle tracking (SPT) [20]. In addition, the penetration of SIV was studied by the use of a mucus layer on which an appropriate amount of virus particles was added. The microscopic diffusion and macroscopic translocation were evaluated. Next, the effects of neuraminidase on the virus mobility in mucus were examined.

## Materials and Methods

### Mucus sample collection

Tracheas were collected from 6-month-old pigs which were negative for swine influenza A viruses as shown by a Hemagglutination Inhibition (HI) test. Using of tracheas from euthanized animals was approved by the Ethical and Animal Welfare Committee of the Faculty of Veterinary Medicine of Ghent University. Two days before euthanization, the pigs were treated intramuscularly with ceftiofur (Naxcel, Pfizer-1 ml/20 kg body weight) to clear the respiratory tract from possible bacterial infections. The tracheas were dissected and the mucus was gently scraped with a spoon, collected with a syringe, and the mucus samples were stored separately at −70°C until use. These samples were negative for neuraminidase determined by NA assay using fluorescent NA substrate (4-methylumbelliferyl-N-acetylneuraminic acid [MU-NANA]).

### Fluorescence lectin staining of α2,3- and α2,6-SA in mucus

Freshly collected mucus was filled in a gelatin capsule (2.3 cm × 0.8 cm), snap frozen in methocel (Fluka). Cryosections of 12 µm were made with a trimming interval of 400 µm between each section. The mucus sections were blocked in 1% (w/v) bovine serum albumin for 1 h, followed by incubation with biotin conjugated *Sambucus nigra* lectin (SNA-I) (EY laboratory, CA, USA; 1∶100) for 1 h, at room temperature. After 2 washings in phosphate buffered saline (PBS), the sections were incubated with Streptavidin-Texas Red (Invitrogen, 1∶200) and FITC labeled *Maackia amurensis* lectin (MAA) (EY laboratory, CA, USA; 1∶25) for 1 h, at room temperature. Afterwards, the sections were washed, and mounted in 90% glycerin containing 2.5% 1,4-diazobicyclo-(2,2,2)-octane (DABCO). Images of the fluorescence staining were acquired using a confocal microscope (Leica TCS SP2 Laser scanning spectral confocal system, Leica microsystems GmbH) and the fluorescence signal was analyzed with ImageJ. The coverage of either SA was calculated by ratio of fluorescence signal to the region of interest (ROI).

### Cells and virus

Madin Darby Canine Kidney (MDCK) cells were maintained in Minimum Essential Medium (MEM, Gibco) supplemented with 10% fetal calf serum (Gibco), 100 µg/ml of streptomycin and 100 units/ml of penicillin (Invitrogen). The avian-like H1N1 swine influenza virus Sw/Belgium/1/98 was used at the third passage on MDCK cells. The virus was propagated in MDCK cells in MEM supplemented with 5 µg/ml trypsin (Gibco), 100 µg/ml of streptomycin and 100 units/ml of penicillin (Gibco).

### Purification of SIV

Confluent MDCK cells were inoculated with SIV at a multiplicity of infection (m.o.i) of 0.01 in MEM. Twenty hours post inoculation, the supernatant was harvested. The cellular debris was removed by ultracentrifugation at 7 000 ×g for 20 min at 4°C in a Type 35 rotor (Beckman, Fullerton, CA, USA) and the suspension was clarified by filtration with a 0.45 µm filter (Millipore). Afterwards, the virus was pelleted at 75 000 ×g for 2 h at 4°C in a type 35 rotor. Following resuspension in PBS (1/100 of the original volume), the virus suspension was brought on a discontinuous OptiPrep (Sigma) gradient containing 10–30% (w/v) of iodixanol and centrifuged at 100 000 ×g for 3 h at 4°C in an SW41Ti rotor (Beckman, Fullerton, CA, USA). The visible opalescent virus bands at the interfaces were harvested separately. The buffer was exchanged with HNE (5 mM HEPES, 150 mM NaCl, 0.1 mM EDTA, pH 7.4) buffer by the use of a 50 kDa filter device (Millipore).

### Verification of the viral purity by lipophilic labeling and immunofluorescence staining of SIV antigens

To verify the purity of the virus from each band, double staining was performed by using 3,3′-Dioctadecyloxacarbocyanine perchlorate (Dio) and hyperimmune swine serum directed to influenza Sw/Belgium/1/98 virus, followed by Texas Red-conjugated goat anti-swine IgG antibody. Hyperimmune swine serum was diluted (1∶50) and mixed with the virus suspension (1∶1, v/v). After 2 h incubation on ice, Texas Red-conjugated goat anti-swine IgG secondary antibody (1∶50, Invitrogen) was added and incubated further on ice for 2 h. Afterwards, the resulting mixture containing virus, hyperimmune serum, and secondary antibody was equilibrated to room temperature. Dio solution (1 mM in DMSO) was mixed with virus suspension (1∶100, v/v) by fierce vortexing, followed by incubation at room temperature for 20 min. The resulting suspension was ultracentrifuged at 100 000 ×g for 1.5 h in a type 35 rotor to remove the free antibodies and dyes. After resuspension in PBS, the virions were clarified by a further ultracentrifugation at 100 000 ×g for 1.5 h. The staining was analyzed with confocal microscope by randomly selecting 10 regions. The ratio of viral antigen positive particles (virions) versus Dio positive particles was calculated, which is referred to as the degree of viral purity.

### Characteristic analysis of the Dio-labeled SIV

The band containing the most purified virus was obtained and the buffer was exchanged with HNE buffer. After incubation with Dio solution as previously described, the virus suspension was filtered by the use of a Sephadex G-50 column (GE Healthcare, Belgium) to remove unbound dye. The unlabeled virus that was eluted through the Sephadex G-50 column was used as negative control. Dio is a lipophilic dye which integrates into the lipid components of the viral envelope. To determine that the integration of the Dio dye into the viral envelope does not change the biophysical properties of the virus, the size and surface charge (*zeta* potential) of the labeled and unlabeled virions were measured by dynamic light scattering and laser Doppler anemometry as previously described [20]. Infectivity and hemagglutination activity of the Dio-labeled viral particles were tested by virus titration and HA assay as previously described [21]. The NA enzymatic activity was determined according to the protocol adapted from Adamo *et al.* [22]. Briefly, 25 µl of fluorescent NA substrate (4-methylumbelliferyl-N-acetylneuraminic acid [MU-NANA], 100 µM in PBS, pH 7.4) was added to 25 µl of each sample containing 16 HA units. After 1 h incubation at 37°C, reactions were stopped with 0.1 M glycine (pH 10.7) in 25% ethanol. Controls and standards were run in parallel, and the fluorescence was measured on a Victor V (Perkin Elmer, Waltham, MA) at an excitation of 360 nm and an emission of 430 nm for 0.1 s per well.

### Microscopic diffusion of SIV in mucus determined by SPT

The trajectories of fluorescent viral particles in porcine tracheal respiratory mucus were recorded by a fast and sensitive electron-multiplying charge-coupled device (EMCCD) camera (Cascade II: 512; Roper Scientific, Tucson, AZ, USA) mounted on an inverted epifluorescence microscope (Nikon TE2000E, Nikon Belux, Brussels, Belgium) equipped with a 100× oil-immersion objective (Plan Apochromat, Nikon). Tracking experiments were performed in press-to-seal silicone isolators (20 mm diameter, 0.5 mm deep, Invitrogen, Merelbeke, Belgium). Our previous study shows that PRV was highly immobilized while 100 nm PGEylated beads were diffusive in porcine respiratory mucus, hence GFP-PRV was used as a negative control and the latter was used as a positive control [20]. GFP-PRV was semi-purified according to the previous protocol [20]. Three microliters of SIV (10^8.5^ TCID_50_/ml) or PRV (10^8.5^ TCID_50_/ml) suspension or 100 nm PEGylated beads (0.004%, w/v) were mixed with 100 µl of porcine tracheal respiratory mucus by gentle stirring. To determine if NA would affect SIV diffusion, the virus was added to the mucus with or without the presence of 0.02 µM zanamivir (Sigma) or 10 mU/ml *Arthrobacter ureafaciens* neuraminidase (Roche Applied Science). The mixture was placed in a custom made glass chamber. The samples were incubated at 37°C for 10 min on the microscope using a stage-top incubator (Tokai Hit, Fujinomiya, Japan) before SPT measurement. Movies were captured with the NIS Elements AR software (Nikon) at a temporal resolution of 33 ms for 5 s. The illumination time was 30 ms per frame. Trajectories of n ≥ 500 particles were analyzed for each experiment and three independent experiments were performed for each condition. Movies were analyzed with the Image Processing Software (IPS, in-house developed software) [23] to extract x, y positional data over time. The apparent diffusion coefficient (*Da*) was calculated as a function of the time scale (*t*) for each particle. Analysis of the movies was performed with IPS. The centroids of individual particles were identified in each frame of a movie. Based on the positions of the centroids, the trajectories of the particles can be determined by a nearest neighbor algorithm. The apparent diffusion coefficient *Da* corresponding to the first time lag *Δt* was calculated according to the classical formula: *Da*  =  MSD/4*Δt* [24]. Afterwards, the distribution of diffusion coefficient of the particles was obtained by maximum entropy method (MEM) analysis [25].

### Penetration of SIV in porcine respiratory mucus

**Figure 1:**
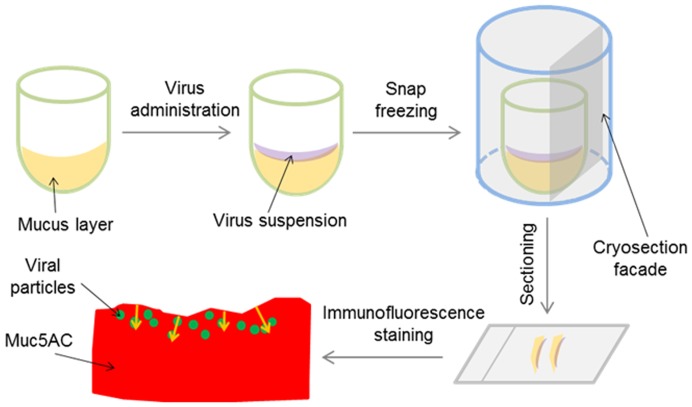
Schematic procedure of virus in-capsule-mucus penetration system. (1) 150 µl of mucus were brought at the bottom of a gelatin capsule. (2) 8 µl of SIV suspension were added on top of the surface of the mucus. (3) Mucus together with virus was embedded and snap-frozen. (4) Cryosections were made vertically to the mucus surface. (5) Immunofluorescence staining was performed to visualize the Muc5AC (representing the mucus) and viral particles. (6) Penetration depth (shown by yellow arrows) was measured from the surface of mucus to the furthest point of the viral signal.

Freshly collected mucus sample (150 µl) was added to a gelatin capsule (2.3 cm × 0.8 cm) to create a mucus “layer” at the bottom (referred to as virus in-capsule-mucus penetration system, Fig. 1). Afterwards, eight microliter of virus suspension containing approximately 10^6.5^ TCID_50_ purified SIV were brought in the form of 5 droplets onto the surface of the mucus. Immediately, 10 min and 30 min after virus addition, the capsules were snap frozen in methocel. Due to a delay of freezing process, the time point “immediately after addition” was designated as 2 min. To determine if the NA would influence the SIV penetration, the virus was added with or without the presence of 0.1 µM Zanamivir (Sigma) or 50 mU/ml *Arthrobacter ureafaciens* neuraminidase onto the mucus, followed by snap-freezing at 30 min post virus addition. Cryosections of 12 µm were made with a trimming interval of 400 µm between each section. Double immunofluorescence staining was performed using mouse anti-Muc5AC IgG1 (45M1, LifeSpan Biosciences, 1∶100) and mouse anti-NP IgG2a (HB-65, ATCC, 1∶50) monoclonal antibodies, followed by Texas Red conjugated goat anti-mouse IgG1 and Alexa Fluor 488 conjugated goat anti-mouse IgG2a secondary antibodies (Invitrogen), respectively. Ten sections were made for each condition, then 10 images were taken, and finally translocations of the virions was measured, as shown in Fig. 1, from the top of the mucus layer until the deepest point of the viral signal.

### Effects of NA on SIV binding to porcine respiratory mucus

Mucus sections (12 µm) were made, and incubated with 30 µl suspension containing 10^6^ TCID_50_ SIV in the presence or absence of 0.1 µM zanamivir or 50 mU/ml *Arthrobacter ureafaciens* neuraminidase at 37°C, for 1 h. The sections were then fixed with 4% paraformaldehyde for 20 min, followed permeabilization in 0.1% (v/v) Triton X-100 for 10 min. Immunofluorescence staining was performed using a mouse IgG antibody to SIV NP, followed by Goat anti-mouse IgG antibody conjugated with FITC to visualize the virions. Fluorescence images were acquired on randomly selected regions with a confocal microscope, and the bound virions on the mucus were calculated with ImageJ.

## Results

### SAs distribution in porcine respiratory mucus

**Figure 2:**
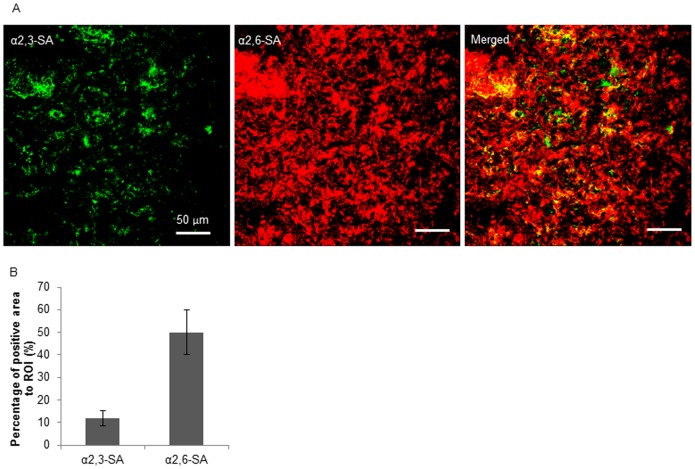
Expression of α2,3- and α2,6-SA on porcine respiratory mucus determined by fluorescence lectin staining. (A) Representative confocal microscopy images. Green color shows α2,3-SA staining and red color represents α2,6-SA staining. The scale bars indicate 50 µm. (B) Semi-quantification of the sialic acids. Three independent mucus samples were analyzed and error bars indicate the standard deviation. The asterisks (**) indicate statistical significance (P<0.01, Student’s *t*-test).

Five sections were examined for each mucus sample for the semi-quantification of α2,3- and α2,6-SA coverage in the mucus. For each section, 2 images were taken and thus 10 images were obtained for each sample. As shown in Fig. 2, the mucus consisted of mixed and heterogeneous α2,3- and α2,6-SA. The SA coverage was calculated by the ratio of the pixels of positive signal to the total pixels measured. The α2,6-SA covered over 50% the region of interest (ROI), while merely 11% of the region was constituted by α2,3-SA.

### SIV purity assessed by double staining

**Figure 3:**
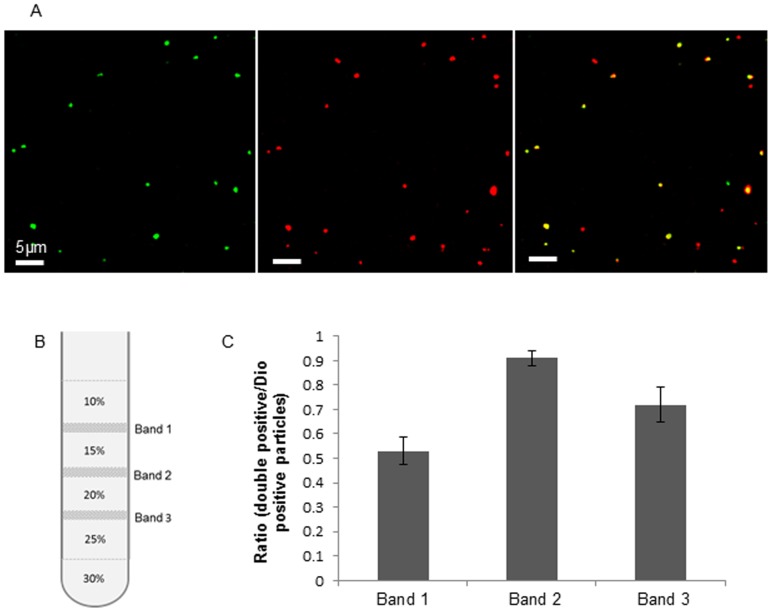
Purity of SIV determined by Dio labeling and immunofluorescence staining. (A) Confocal microscopy of the double staining of the virus preparations. Green represents Dio labeled particles; viral antigens are shown in red. Merged signals represent virus particles which are also labeled with Dio. (B) Bands form in the discontinuous iodixanol gradient separation. Three bands were identified, named Band 1, Band 2 and Band 3 from up downwards. (C) Ratio of double positive particles versus Dio positive particles for the particles from three different bands. Three independent experiments were performed and error bars indicate the standard deviation.

After ultracentrifugation over a discontinuous OptiPrep gradient containing 10% to 30% of iodixanol, three visible opalescent bands were collected, named Band 1, Band 2 and Band 3, respectively, from top to bottom (Fig. 3B). The purity of virus from each band was assessed by confocal microscopy following Dio lipophilic dye labeling and SIV immunofluorescence staining. As Dio integrates into the lipophilic components of virus and cellular debris, Dio staining was used as a total-particle assessment. The red color visualized the viral particles, and the green color represented Dio-labeled particles (Fig. 3A). The percentage of double positive particles versus Dio positive particles represents the virus purity in a ratio extent. Consequently, the highest viral purity (over 0.9 for the ratio of double positive particles/Dio positive particles) was found in Band 2 (Fig. 3C). Therefore, the virus preparation from Band 2 was used for further analysis.

### Characterization of Dio-labeled SIV

After incubation with 10 µM Dio dye at room temperature, followed by elution in a Sepharose G-50 column, the labeled and unlabeled SIV were analyzed for different characteristics. The results show that the hemagglutination activity and infectivity were not altered by labeling. The neuraminidase activity of Dio-labeled SIV was 91% of that of unlabeled SIV. Measured by dynamic light scattering and laser Doppler anemometry, the size and surface charge of the labeled virions were not significantly altered (Table 1).

**Table 1:** Characteristics of SIV and Dio-labeled SIV.

	Zeta potential (mV)	Diameter (nm)	Infectivity (TCID_50_ lg/ml)	HA titer	NA activity (RFU*)
SIV	−24.2±3.8	101.8±2.9	8.7±0.43	256	34017±3250
Dio-Labeled SIV	−28.1±5.1	113.5±2.5	8.5±0.23	256	30950±4172

*RFU =  Relative Fluorescence Units.

### SIV was partially diffusive in porcine respiratory mucus

**Figure 4:**
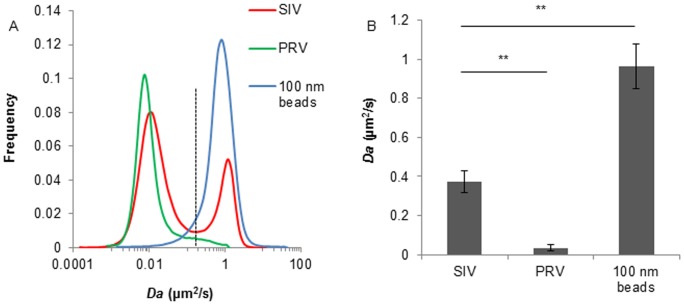
Diffusion coefficient of SIV in mucus and comparison with PRV and 100 nm PEGylated beads. (A) Distributions of the apparent diffusion coefficient of SIV, PRV and 100 nm PEGylated beads in porcine respiratory mucus. Trajectories of 8 steps were analyzed for each of the 1500 diffusion coefficients. Distributions were refined with MEM. Dashed line indicates the boundary of mobile and immobile diffusion. (B) Average diffusion coefficient of SIV, PRV and 100 nm PEGylated beads. Data were obtained from three independent experiments, and error bars indicate the standard deviation. The asterisks (**) indicate statistical significance (P<0.01, by Student’s *t*-test).

The motion of SIV in porcine respiratory mucus was investigated and compared with the diffusion of PRV and 100 nm PEGylated beads. Trajectories of 8 steps were analyzed, from which a distribution of the apparent diffusion coefficients was obtained. Similar to our previous data, PRV was highly hindered in the porcine respiratory mucus, while the 100 nm PEGylated beads diffused freely. The distribution of diffusion coefficient clearly demonstrated that, compared to one immobile fraction for PRV or a mobile fraction for the 100 nm beads, SIV experienced two diffusion patterns in porcine respiratory mucus, with 70% of viral particles being trapped while the rest of particles moving rapidly (Fig. 4A). The average diffusion coefficient of SIV in mucus was 11-fold higher than that of PRV (Fig. 4B). The similar size 100 nm PEGylated beads are muco-innert which indicates that these particles did not interact with any type of the mucus moieties. Thus to the opposite, the viral particles were immobilized probably due to binding interactions with the mucus. These data suggest that binding and releasing effects were present in the interactions of SIV with porcine respiratory mucus.

### Penetration of SIV through the mucus layer

**Figure 5:**
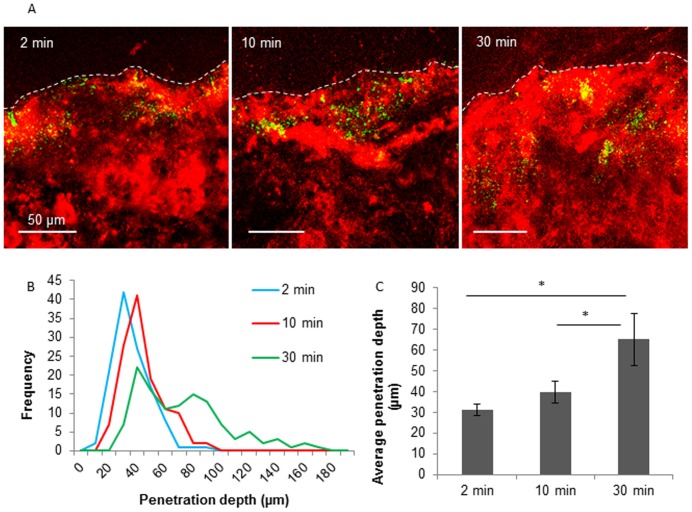
Penetration of SIV through porcine respiratory mucus. (A) Confocal microscopic analysis of the virus penetration. Representative confocal photomicrographs of the penetration of SIV at 2, 10 and 30 min post virus addition. Mucin 5AC and SIV antigens were visualized by red and green color, respectively. (B) Penetration depths of SIV with time. Hundred and twenty measurements were obtained from three independent mucus samples. (C) Average penetration depth of SIV at 2, 10 and 30 min post virus addition. Three independent experiments were performed. Error bars indicate the standard deviation. The asterisk (*) indicates statistical significance (P<0.05, by Student’s *t*-test).

The depth of SIV penetration could be visualized by double immunofluorescence staining of the Muc5AC and SIV nucleoprotein (NP). The distance from the surface down to the deepest point of virus translocation was measured and designated as the depth of virus penetration (Fig. 1). Three independent experiments were performed and in total 120 measurements were conducted. Distribution of the penetration depth for each condition was eventually obtained. Immediately after virus addition, the virions rapidly entered the mucus layer and reached a depth of 31 µm within 2 min, due to a passive diffusion effect (Fig. 5A). Incubated at 37°C, the virions spread further in the mucus with time. The distribution of penetration depth shows that the majority of SIV particles travelled 10 µm further in the mucus from 2 until 10 min after virus addition and reached a depth of up to 180 µm at 30 min after addition (Fig. 5A). Similarly to the microscopic diffusion, the distribution of SIV penetration clearly shows two fractions at 30 min after virus addition (Fig. 5B). About 65% of the viral particles penetrated at 30 min more than 2-fold further than 2 min post virus addition (Fig. 5B). The average depth of virus penetration at 30 min was significantly higher than that of earlier time points (Fig. 5C), suggesting that the SIV virions were able to actively penetrate the mucus layer.

### Neuraminidase mediated the diffusion and penetration of SIV in respiratory mucus

**Figure 6:**
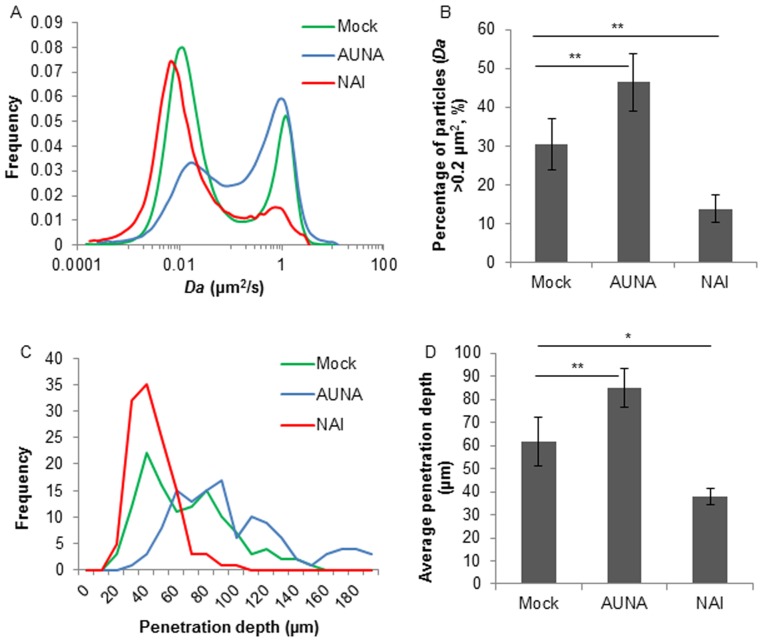
Effects of NAI and *Arthrobacter ureafaciens* neuraminidase (AUNA) on microscopic diffusion and macroscopic penetration of SIV in porcine respiratory mucus. (A) Distribution of diffusion coefficient of NAI, AUNA and mock treated SIV in mucus. 1500 trajectories were analyzed. Distributions were refined with MEM. (B) Proportion of mobile fraction (*Da*>0.2 µm^2^/s) of NAI, AUNA and mock treated SIV in mucus. Error bars represent the standard deviation from three independent experiments. The asterisks (**) indicate statistical significance (P<0.01, by Student’s *t*-test) (C) Distribution of the penetration depth of NAI, AUNA and mock treated SIV through mucus at 30 min post virus addition. Hundred and twenty measurements were performed on three independent mucus samples. (D) Average penetration depth of NAI, AUNA and mock treated SIV through mucus at 30 min post virus addition. Three independent samples were performed, and error bars indicate the standard deviation. The asterisks (** and *) indicate statistical significance (P<0.01, and P<0.05, respectively, by Student’s *t*-test).

Movies were captured with SPT software, and the SIV microscopic diffusion in mucus in the presence or absence of zanamivir or exogenous neuraminidase was analyzed with IPS. As shown in Fig. 6A, the mobile fraction of SIV diffusion was severely diminished by NAI treatment, while was elevated by the addition of *Arthrobacter ureafaciens* neuraminidase. Approximately 55% of the mobile viral particles (with *Da* larger than 0.2 µm^2^/s) became stuck by the effect of zanamivir whereas the exogenous neuraminidase increased the mobile particles by approximately 15% (Fig. 6B). Consistently, the presence of zanamivir in virus suspension almost completely inhibited the SIV macroscopic penetration which contrasts the further penetration by the effect of exogenous neuraminidase (Fig. 6C). The average penetration of mock treated SIV was significantly higher than that of NAI treated virus, while the rise of average penetration from mock to neuraminidase treated virus was also significant (Fig. 6D). These data imply that neuraminidase helped SIV penetrate through the porcine respiratory mucus.

### Effects of NA on SIV binding to the porcine respiratory mucus

**Figure 7:**
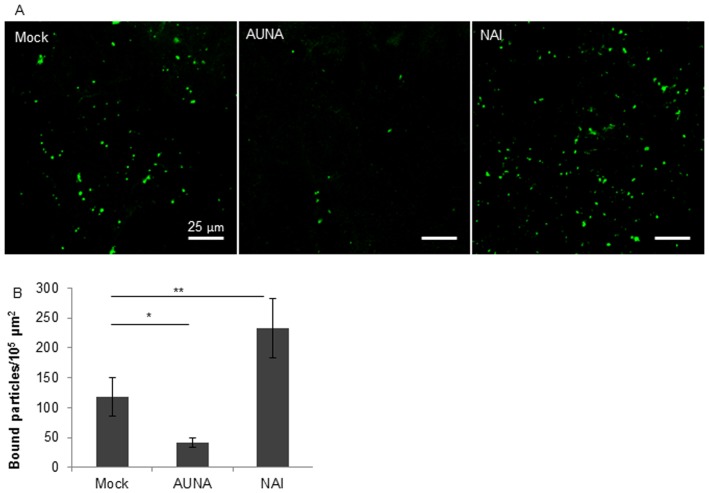
Effects of NA on SIV binding to porcine respiratory mucus shown by confocal microscopy. (A) Virions (green) bound to mucus sections in the presence or absence (Mock) of Zanamivir (NAI) or *Arthrobacter ureafaciens* neuraminidase (AUNA). (B) Quantification of viral particles bound to mucus (per 10^5^ µm^2^). The error bars indicate the standard deviation from 3 independent experiments. The asterisks (** and *) show the significance difference (P<0.01, and P<0.05 by Student’s *t*-test).

Virus attaching to the mucus sections was visualized by immunofluorescence staining to the SIV NP. The virus binding to 5 mucus sections was analyzed, 2 images were taken for each section and in total 10 images were obtained for virions quantification. The virions that attached to a mucus region of 10^5^ µm^2^ were calculated. Three independent experiments were performed. The representative confocal photomicrographs show that zanamivir clearly enhanced the attachment of SIV to the mucus. In contrast, the exogenous neuraminidase depleted the virus binding to the mucus by 2-fold (Fig. 7). These data clearly demonstrated that NA was able to release the SIV particles which may have been bound by interaction of HA with mucins, moving the virions through the mucus.

## Discussion

Influenza viruses are highly contagious and readily spread by aerosol transmission. The mucus is the first barrier for the small aerosol droplets to settle and overcome. In the present study, we applied SPT technique and a custom made virus in-capsule-mucus penetration system to visualize the microscopic diffusion and macroscopic penetration of SIV in porcine respiratory mucus.

SPT is a unique model for rigorous analysis of virus-mucus interactions from the mobility point of view. The virus in-capsule-mucus penetration system allows the visualization of virus penetration in mucus layer thereby mimicking the natural conditions. By the use of these models, we were able to track the diffusion of SIV in natural respiratory mucus. In the SPT assay, there were two fractions based on the virus diffusion coefficient, a mobile and an immobile fraction. The ability of SIV to detach from mucus was attributed to the NA activities, as inhibiting NA by the use of zanamivir significantly suppressed the liberation of the virus from the mucus network (Fig. 6A, 6B). This is also in line with a previous report by Matrosovich *et al* [26], which describes that blocking of the NA activities by oseltamivir efficiently inhibited influenza A viruses from infecting the differentiated human airway epithelium cultures which were probably covered by mucin secretions. Furthermore, exogenous neuraminidase was shown to promote both the microscopic diffusion and macroscopic penetration detected by the SPT and virus in-capsule-mucus penetration system (Fig. 6). This does not only confirm the beneficial effect of neuraminidase on releasing SIV from respiratory mucus, but also highlights bidirectional synergistic interactions between influenza virus and bacterial infections. The influenza virus predisposition to secondary bacterial infections has been thoroughly studied [27], [28], however, little information exists regarding the impact of bacterial neuraminidase on influenza virus entry and transmission, and further research is needed.

Neuraminidase was indicated to play a role in the SIV releasing from mucus, however, two fractions of viral motion in the mucus lead to a discussion of the way that the virus binds to the mucus. Mucins are the major constitute of mucus and are highly decorated by glycans terminated by sialic acids, thus they are likely to be attributed to the immobilization of SIV in porcine respiratory mucus. Mucins may play direct and indirect roles in host defense distinct from their ability to form adhesion decoys. In addition to mucins, the aqueous mucus layer consists of a great number of host defense agents including lysozyme, lactoferrin, secretory IgA (sIgA), collectins, defensins, cathelicidins, histatins and surfactant proteins [29]–[31]. These molecules may function by binding the virions in a receptor-independent pattern. It has been shown that surfactant protein D (SP-D) binds via a carbohydrate recognition domain in a Ca^2+^-dependent manner to N-linked high-mannose carbohydrates present on the HA and NA of the influenza viruses [32]. In addition, sIgA is retained at high concentrations in mucus where it can efficiently trap the pathogens. Last but not least, defensins are of great interest with respect to respiratory viral infection. Human defensins have been shown to bind several types of viruses and inhibit the entry of the viruses to target cells [33], [34]. These components may be retained in mucus by direct binding with mucins or by the biophysical properties of mucus and thus become part of the gel network and provide an immobilized reservoir of protective effectors. Alternatively, it is possible that the viral particles that were immobilized in the mucus were actually incomplete or defective virions or became inactive while being prepared. The production of defective particles has been reported for influenza A viruses [35], [36]. However, the approach of distinguishing active and inactive virions in an entity has not been readily achieved, and will be further examined.

While vaccination remains the primary option for the prevention and control of influenza, anti-influenza virus drugs are considered as a complementary approach, as vaccine production may not be rapidly achieved. Our findings provide experimental evidence for the essential role of NA in influenza virus penetration through the respiratory mucus. Blocking the NA activities clearly suppressed the movement of virus in mucus (Fig. 6), illustrating that NA played a role in removing the sialic acids on mucins, which may enable the virus to gain access to the cellular receptors. This suggests the usefulness of neuraminidase inhibitors as prophylactic treatment for influenza. On the other hand, preventative treatment with oseltamivir (Tamiflu) failed to protect the lung from virus replication or inflammation in an *in vivo* influenza infection study in pigs despite reduced clinical symptoms and virus shedding [37]. This highlights the complexity of the *in vivo* situation and the minimal benefits neuraminidase inhibitors may have.

The ability of an influenza virus passing through the mucus may serve as a determinant for influenza virus transmission in addition to efficient virus attachment, high potential of replication and low infectious dose required [5], [38], [39]. Combining the study of Cohen *et al.* [19], it can be noticed that human influenza viruses could bind and be released from human salivary mucins but not from porcine submaxillary mucins, whereas, swine influenza virus was able to escape from porcine airway mucus, suggesting there may be different interactions between different influenza viruses and the mucus of different species. A balance of binding to and releasing from the mucin sialic acids, which is determined by the functional balance of HA and NA, may influence how efficiently the virus avoids sticking to mucus. Fluorescence lectin staining on mucus cryosection showed that both α2,3- and α2,6-SA were present in the porcine respiratory mucus, with distinct predominance for the latter (Fig. 2). The binding profile of the SIV strain was not investigated in this study, however, it has been well documented that swine influenza virus isolates, especially those with the avian-like H1 and H3 hemagglutinins showed receptor specificity for both α2,3- and α2,6-sialylated glycans [40]–[42]. Probably the mucus provides sufficient amount of receptors for SIV binding. The binding of SIV via HA to the porcine respiratory mucus was proved in the present study, and the amount of viral or exogenous NA indeed modulated the extent of viral binding to and releasing from the porcine mucus (Fig. 7). Concerning the releasing effect, NA which mediates the process also has a substrate preference. It was demonstrated that NA of human and swine influenza viruses have a preferential specificity for α2,3-SA although they cleave both linked sialylated glycans [43], [44]. Therefore, we assume that the sialic acids in respiratory mucus secretions may exert an effect on influenza virus transmission.

Since the majority of viral particles were incapable of penetrating through the mucus layer, why do influenza viruses invade the respiratory tract of the animals after all [1], [3], [45]? Based on our experimental findings and present literature, we propose several strategies the influenza viruses may use to overcome the mucus barrier and find their way to establish infection:

1. Production of enzymes that aid the virus movement through the mucus. Influenza virus binds to and uses sialic acid-containing molecules as receptors. It is because of this capability that influenza virus has evolved a second viral surface protein, neuraminidase, as a receptor-destroying enzyme that cleaves sialic acid, allowing the virus to be released after binding to sialic acid-containing molecules that do not lead to viral infection. This strategy is utilized as well by many other microbes, such as *E. histolytica* [46], [47], *Vibrio cholerae* [48], *Helicobacter pylori* [49], Reovirus [50] and Coronavirus [51], to subvert or avoid the mucus barrier. The production of enzymes, including mucinase, sialidase, glycosidase, elastase, and hydrolase, which are capable of degrading mucin core proteins and mucin carbohydrates facilitates microbes to swim through the mucus layer. Furthermore, the enzymes that the microbes produce may also facilitate the invasion of other pathogens. In women with bacterial vaginosis, the overgrowth of anaerobic gram-negative bacteria that produce sialidase, glycosidases and other mucin-degrading enzymes causes a breakdown in the barrier properties of cervicovaginal mucus, thereby destroying the mucus gel and helping other sexually transmitted pathogens such as human immunodeficiency virus (HIV) to invade [52].
2. The use of abundant and ubiquitous molecules as receptors. Although there may be a risk of binding to decoy receptors, the use of abundant and ubiquitous molecules as receptors provides the apparent advantage to the virus for allowing infection of multiple cell types and species. This can result in a low minimal infectious dose for initial infection. Based on the data of diffusion and penetration, the effects of the mucus network that virions encounter are so extreme that only a part of the particles can escape and reach susceptible target cells ultimately. Thus the viruses which require lower minimal infectious doses for the same tissues may gain higher chance to establish an infection.
3. Spreads via aerosol. The slow settle of aerosols in the air can cause prolonged contact of the virus with the respiratory tract which benefits the virus penetration through the mucus layer. Furthermore, aerosol droplets can travel much more efficiently to the lower respiratory tract and the mucociliary apparatus may need a longer time to transport and exclude the virions out of the respiratory tract, which increases the chance of these viruses to penetrate through the mucus layer and reach the target cells eventually.

The issues on if SIV would be able to penetrate through the porcine respiratory mucus and if the neuraminidase would contribute to move the virus through the mucus layer have been addressed. However, the ability of the viral neuraminidase to cleave sialic acid from mucus has not been investigated due to technical limitation. The viscous property impedes the separation of the free sialic acids from the mucus even if they would have been cleaved by the viral neuraminidase. Investigating the role of influenza virus neuraminidase in the cleavage of sialic acid from mucus may shed some light on unravelling the mechanism of influenza pneumonia. Hence the effect of influenza virus neuraminidase on mucus needs to be studied.
